# *Treponema pallidum* infection in asymptomatic persons: A puzzling scenario in the Canary Islands (Spain) (2001–2020)

**DOI:** 10.1371/journal.pone.0325073

**Published:** 2025-07-08

**Authors:** Jose Luis Pérez-Arellano, Araceli Hernández Betancor, Oscar Sanz Peláez, Jose Curbelo, Michele Hernández Cabrera, Elena Pisos Álamo, Nieves Jaén Sánchez, Laura Suárez Hormiga, Carmen Lavilla Salgado, Laura López Delgado, Sandra González Linares, Cristina Carranza-Rodríguez

**Affiliations:** 1 University Institute of Biomedical and Health Research (Instituto Universitario de Investigaciones Biomédicas y Sanitarias IUIBS), University of Las Palmas de Gran Canaria (ULPGC), Spain; 2 Microbiology and Parasitology Service, Insular University Hospital of Gran Canaria, Las Palmas de Gran Canaria, Las Palmas, Spain; 3 Department of Medical and Surgical Sciences, University of Las Palmas de Gran Canaria (ULPGC), Spain; 4 Unit of Infectious Diseases, University Hospital of Gran Canaria Dr. Negrín, Las Palmas de Gran Canaria, Las Palmas, Spain; 5 Department of Medicine, University Francisco de Vitoria, Madrid, Spain; 6 Unit of Infectious and Tropical Diseases, Insular University Hospital of Gran Canaria, Las Palmas de Gran Canaria, Las Palmas, Spain; 7 Canary Institute of Hemodonation and Hemotherapy,; 8 University of Las Palmas de Gran Canaria, Las Palmas de Gran Canaria, Las Palmas, Spain; Universidade Federal do Espirito Santo, BRAZIL

## Abstract

**Background and objectives:**

Syphilis is an infectious disease caused by *T. pallidum* subsp. *Pallidum*. In high-income countries the main mode of transmission is sexual. Approximately half of infected patients are asymptomatic, which does not exclude the possibility of transmission. The aim of this study was to evaluate syphilis seroprevalence among asymptomatic persons in Gran Canaria (Canary Islands, Spain).

**Patients and methods:**

Three different groups were studied from 2001 to 2020*: i)* a “blood donor” sample of 948,869 voluntary blood donations as a proxy of health population.; *ii)* undocumented African immigrants, including 1,873 recent arrivals in Gran Canaria; and *iii)* people living with HIV (PLWH), a group of 1,690 patients followed by our team. The evaluation included both treponemal and reaginic tests.

**Results:**

*i)* among blood donors, the mean seroprevalence of positive treponemal tests was 0.25% (95% CI: 0.19–0.31). Non-treponemal test positivity (RPR) ranged from 0.05 to 0.06% with titers ≤ 1:4 in all cases; *ii)* thirty-four of 641 undocumented African migrants (5.30%; 95% CI: 3.82–7.32%) had a confirmed positive treponemal test but only 4 had a positive RPR, with titers ranging from 1:1–1:4; *iii)* 46.51% (95% CI: 44.14–48.89) of PLWH patients had a confirmed positive treponemal test. For factors related to HIV-syphilis coinfection, multivariate analysis clearly showed the association with male sex and the MSM risk category. However, the results of this series call into question the overall role of immigration in the seroprevalence of syphilis among PLWH in our setting. Active syphilis (RPR > 1:8) was found in 20.10% of PLWH.

**Conclusions:**

In summary, syphilis is a re-emerging infection, and asymptomatic persons constitute a group that facilitates its transmission and spread. In our setting, seroprevalence was lowest in blood donors, higher in recently arrived African migrants, and highest in PLWH, especially MSM. The presence of active syphilis however is mainly restricted to MSM. This information is of relevance for the design of syphilis control strategies.

## Introduction

Syphilis is a systemic disease caused by spirochetes of the species *Treponema pallidum* subspecies *pallidum* [1–3]. Other species that can infect humans are *T. pallidum* subsp. *pertenue*, the causative agent of yaws, *T. pallidum* subsp. *endemicum*, responsible for endemic syphilis (bejel), and *T. carateum*, which causes pinta [3], with a more limited geographical distribution. The most common mode of transmission of *Treponema pallidum* subspecies *pallidum* is direct contact between an infected individual with cutaneous or mucosal lesions and a healthy individual, typically through sexual contact. Other routes of transmission are transplacental, responsible for congenital syphilis, and through contact with infected blood (through transfusions or the sharing of syringes) [1–3].

The natural history of syphilis has several clinical phases [1–3]. Following an incubation period of 9–90 days, the initial symptoms of primary syphilis appear, typically locally (chancre and lymphadenopathy). Appropriate treatment results in the disappearance of the lesions and the prevention of dissemination. In the absence of treatment, however, the lesions will disappear spontaneously, but without preventing dissemination. [1,2] After 2–8 weeks, the clinical manifestations of secondary syphilis, corresponding to hematogenous spread, may become apparent. These manifestations can affect virtually the entire body (including the skin, scalp, lymph nodes, liver, meninges or kidneys). As with primary syphilis, the lesions will disappear with appropriate treatment, although they may also occur spontaneously within weeks [2]. The subsequent phase, latent syphilis, has no clinical manifestations, and is divided into two periods: the early latent phase (up to one or two years after acquisition) and the late latent phase (after this period or when the time cannot be determined) [1,3]. Approximately one third of patients in the untreated latent phase develop tertiary syphilis, with neurological manifestations or cardiovascular and gummatous infections [2,3]. An important aspect of epidemiologic relevance is that up to 50% of patients with syphilis have no clinical manifestations (asymptomatic syphilis) [3], which can be attributed to two main factors: a mucosal location that is more difficult to visualize (such as the vagina, oropharynx or rectum) and the typically painless nature of the lesions.

The incidence of syphilis, known since ancient times, showed a stable, even decreasing trend until the beginning of the twenty-first century [4]. Since then, however, there has been a notable increase in syphilis, both worldwide and in Europe and Spain [5]. In 2022, the WHO reported 8 million cases of syphilis worldwide [6]. In the EU/EEA (European Union/European Economic Area), the latest report of the ECDC (European Centre for Disease Prevention and Control) showed a confirmed syphilis rate of 8.5 cases per 100,000 inhabitants, with Spain being the country with the second highest number (after Germany) [7]. A temporal trend analysis in Spain identified four distinct periods: 1995–2001, with a downward trend; 2001–2011 with an upward trend, 2011–2014, when it stabilized, and from 2014 onwards, another increases greater than the previous one [8].

It is likely that epidemiologic data on clinical syphilis are underestimated and vary depending on the population studied. Some series include the general population [9–11], while others are limited to specialist consultations, hospitals or prisons [12–16]. The seroprevalence of asymptomatic syphilis is highly variable because it is based on studies conducted in populations without clinical manifestations [17–44] and influenced by multiple factors including age [17], study setting (primary care [18], specialized units [19–23], shelters [24–28] or immigration services [29–31]), the presence of other coinfections, mainly HIV and other sexually transmitted infections (STIs) [32], specific population groups, such as pregnant women [33–36], those at potential risk of acquisition, such as persons who inject drugs (PWID) [37], sex workers [38], men who have sex with men (MSM) [39–41] or have nephropathies [42]. In addition, the region or country of origin of migrant arrivals [18,20,21,43] and length of residence in Spain [44] are important considerations. Finally, the results of the various series vary according to the year of study.

Two specific features of the Canary Islands (Spain), particularly the island of Gran Canaria, may affect the incidence and prevalence of this infection. These are undocumented migrants, mainly from sub-Saharan Africa, and MSM tourism, especially in the south of the island. Both groups are populations with high geographical mobility, which may influence their importance in terms of prevalence and the possibility of transmission.

The objective of our study was to evaluate and compare the seroprevalence of syphilis infection in three different groups of asymptomatic individuals: *i)* Blood donors, *ii)* Recent immigrants from Africa, and *iii*) People living with HIV.

## Patients and methods

This retrospective study of the prevalence of asymptomatic syphilis was carried out in Gran Canaria (Canary Islands, Spain). For the purposes of this study, asymptomatic syphilis was defined as a confirmed serologic treponemal test result in the absence of any previous or current clinical manifestations suggestive of the disease or a history of treatment for this infection. The data for this research were accessed on February 1, 2015, and data collection was completed on March 31, 2024. The authors did not have access to information that could identify individual participants during or after data collection.

### Study groups

Three groups of adults (≥18 years old) were included: a healthy local population; a population consisting of undocumented migrants (from Africa) and a group of people living with HIV (PLWH).

#### Voluntary blood donors.

The inclusion criteria were those used by the Instituto Canario de Hemodonación y Hemoterapia [Canarian Institute of Hemodonation and Hemotherapy] of Las Palmas, which provided the available data on syphilis seroprevalence in blood product donations from January 1, 2001 to December 31, 2020. It should be noted that the data presented here correspond to blood donations (948,869) and not to individuals. The exclusion criteria for donation were as established by law in Spain [45] and were determined after a complete medical history to assess the presence of sexual risk factors or illicit substance abuse, as well as a history of prior infection with HIV, HBV or HCV.

#### Undocumented migrant population.

The inclusion criteria were as follows: a demographic and clinical assessment of persons of African origin who had recently arrived in Gran Canaria, access to these persons, and availability of blood samples obtained from them. Although the planned study period was from January 1, 2001 to December 31, 2020, there was a sharp decrease in the number of undocumented migrants in the Canary Islands between these dates from 2011 onwards Specifically, our study focused on individuals admitted to the UDJAMA (Red Cross) Immigrant Reception Center in Las Palmas de Gran Canaria, which received newly arrived migrants (less than 6 months) over 18 years of age who had been detained by State Security Forces while attempting to enter Spanish territory, pending expulsion or repatriation specifically between 2001 and 2004. These individuals were considered new arrivals and consequently any infections they may have had were specific to their country of origin or had been acquired during the journey, but not in Spanish territory. Following a clinical evaluation and physical examination, biological samples (blood, stool and urine) were requested for complementary tests. In the final analysis, those individuals who refused complementary tests or abandoned the study center before they could be obtained were excluded.

#### People living with HIV.

Inclusion criteria were confirmed diagnosis of HIV infection (before or during the study period) at the Infectious Diseases and Tropical Medicine Unit of the HUIGC, patient follow-up between January 1, 2001 and December 31, 2020) and during follow-up in 2020, as well as clinical evaluation, physical examination and complementary tests. Patients for whom the relevant information was not available were excluded from the study.

### Screening for *Treponema pallidum* infection

All samples were tested for syphilis using treponemal tests. The techniques used varied depending on the date of the study and availability at each center: Trepo-Spot IF (bioMérieux, Spain), Vitros Syphilis TPA assay (Ortho Clinical Diagnostics, Inc., High Wycombe, United Kingdom) and Architec Syphilis TP (Abbott Diagnostics, Spain).

To confirm positive results, a second treponemal test by line immunoassay (LIA) was conducted using the INNO-LIA® Syphilis Score (Fujirebio Iberia, Spain).

In cases where positive results were confirmed, a non-treponemal RPR (rapid plasma reagin) test was conducted using either a commercial RPR test (Human Diagnostics, Spain) or the RPR slide test (bioMérieux, Spain) technique. Nontreponemal tests are useful in monitoring disease activity, considering that RPR titer equal or greater than 1/8 indicates active disease [46,47].

### Other determinations

In blood donors, data on epidemiology, personal history and anamnesis were included. Finally, in addition to the above data, a complete physical examination was performed in an undocumented migrant population and people living with HIV.

Further studies were also performed. Overall, we screened for HIV infection using an enzyme microparticle immunoassay for detection of antibodies to HIV types 1 (groups M and O) and 2 (AxSYM®HIV ½ gO) (Abbott Diagnostics, Spain). To confirm the initial results, we used a strip immunoblot INNO-LIA^TM^HIV (Innogenetics N.V.) that can detect and differentiate antibodies to HIV types 1 (groups M and O) and 2. Enzyme immunoassay techniques (Abbott Diagnostics, Spain) were employed to detect the various markers of HBV infection. First, hepatitis B surface antigen (HBsAg) and antibodies to both the core and surface antigens (anti-HBc and anti-HBs respectively) of the hepatitis B virus were determined. In cases where HBsAg was identified, the presence of HBeAg (Abbott Diagnostics, Spain) and HDV (Dia.Pro, Diagnostic Bioprobes) was determined. For HCV infection, we first used the microparticle enzyme immunoassay, AxSYM HCV version 3.0 (Abbott Diagnostics, Spain) for antibody detection, and a strip immunoblot, INNO-LIA^TM^HCV Ab III update (Innogenetics N.V.) for confirmation.

### Statistical analysis

Data analysis was performed using Stata 13.0. Normality of data was assessed using the Kolmogorov-Smirnov test, and homogeneity of variance using Levene’s test. Categorical data were presented as frequencies and percentages, and continuous data as means and standard deviations (SD) or median and interquartile range (IQR), as appropriate.

Categorical variables were compared using Pearson’s chi-squared test, or Fisher’s exact test when indicated. The strength of the associations was measured using odds ratios (OR), with 95% confidence intervals (95% CIs). For prevalence estimates, confidence intervals were calculated using the Wilson method. A multivariate analysis was performed to study the risk factors associated with syphilis infection, including clinically relevant and statistically significant variables.

Trend analysis of seroprevalence in the study period was performed using the Jointpoint Regression Program version 4.5.01, with annual percentage change (APC) reported as mean and 95% confidence interval. In this study, the minimum number of joinpoints was set at 0, corresponding to a linear model with no change in trend. The maximum number of joinpoints was set at 3, taking into account the number of available data points and following the recommendations of the Joinpoint Regression Program. The final model was selected using the software’s built-in permutation test, which balances model fit and parsimony.

A p-value of less than 0.05 was used to determine statistical significance.

### Ethical aspects

The study was conducted in accordance with the protocol and principles established in the current revised version of the Declaration of Helsinki (Fortaleza, October 2013) and approved by the Research Ethics Committee of the Insular Maternal and Infantile University Hospital Complex (CEIC-CHUIMI-2014/750). The documentation required for approval by the Research Ethics Committee explicitly included the exclusion of the informed consent of the participants due to the retrospective nature of the study and the anonymization/dissociation of their data.

## Results

### Seroprevalence in blood donors

**Fig 1:**
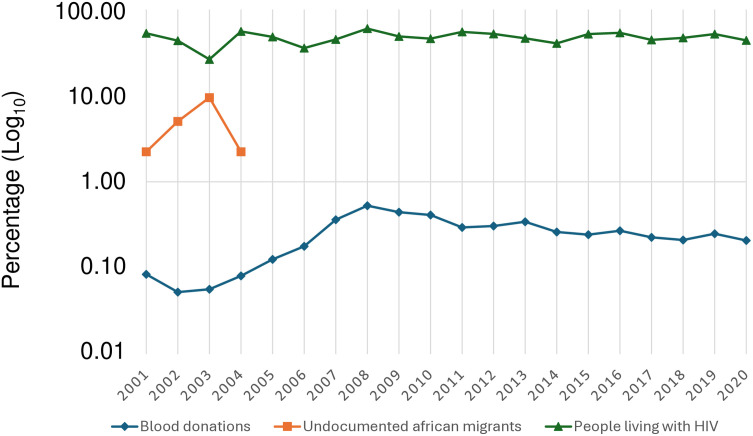
Positive treponemal test percentage in the study groups. Each point indicates the percentage according to the year of study.

Over the course of the study period (2001–2020), a total of 948,869 blood donations were evaluated for the presence of *Treponema pallidum* infection, representing an average of 47,443 per year, with a male-to-female ratio of 1.6:1. The mean seroprevalence of syphilis (positive treponemal test) was 0.25% (95%CI: 0.19–0.31). Fig 1, S1 and S2 Tables illustrates the trend over the study period. While seroprevalence was higher among males and individuals under 35 years of age, no significant differences were observed with respect to females or individuals over that age limit, respectively. Trend analysis showed stabilization during this time period, with an APC of 9.85% (95%CI: −6.82%;26.52%). Non-treponemal test-positive cases (RPR) ranged between 0.05 and 0.06% with titers ≤ 1:4 in all cases.

### Seroprevalence in undocumented migrants

A total of 1,873 undocumented migrants were examined over the course of the study; 68.5% (n = 1,283) were from sub-Saharan Africa. In 3.4% of cases, the country of origin of the subjects was not known. The distribution of migrants by country of origin is shown in S1 Fig, with the highest frequencies (in descending order) being from Morocco, Nigeria, Sierra Leone, Ghana and Mali. The mean age was 26.61 (SD: 6.35) years and 91.1% of the participants were male. There were no statistically significant differences in age between the populations from the North African and sub-Saharan areas.

A total of 641 individuals (S1 and S2 Tables) underwent serologic testing, with 34 cases yielding confirmed positive treponemal test results (5.30%; 95%CI 3.82–7.32%). Of the 34 cases, 4 were RPR positive, with titers ranging from 1:1–1:4.

### Seroprevalence in people living with HIV

A total of 1,690 patients with follow-up in 2020 were studied, using 2001 as year of onset. Age at onset ranged from 8 to 82 years, with a mean of 39 years and a standard deviation of 12 years. Of these, 1,465 (86.7%) were cisgender men,208 (12.3%) were cisgender women and 17 (1.0%) were transgender women. In terms of geographical origin, 1,140 (67.46%) were local and 550 (32.54%) were immigrants. For immigrants living with HIV, the continents of origin were Europe (295/550; 53.6%), the Americas (182/550; 33.1%), Africa (68/550; 12.4%) and Asia/Oceania (5/550; 0.9%). The infection vulnerabilities were identified in 1,550 patients: *i)* 1,104/1,550 (71.2%) were MSM; men who have sex with men (gay and bisexual); *ii)* 340/1,550 (21.9%) were HTS; heterosexuals; *iii)* 97/1,550 (6.3%) were PWID; persons who inject drugs, and *iv)* 9/1,550 (0.6%) were other modes of transmission (transfusion or mother to child).

Among people living with HIV, 46.51% (95%CI: 44.14–48.89) had a confirmed positive treponemal test. Fig 1, S1 and S2 Tables also shows the temporal evolution of seroprevalence among people living with HIV. Trend analysis showed stabilization during this period with an APC of 2.01% (95%CI: −9.81%; 13.83%).

Table 1 shows the demographic characteristics of people living with HIV, both overall and according to *T. pallidum* infection. Statistical analysis showed a significant association between age, sex, infection vulnerabilities, geographical origin (immigrant or local) and region of origin of immigrants. The strength of the association was stronger between the prevalence of *T. pallidum* infection and male sex [OR: 6.5 (4.3–9.8)], MSM infection vulnerabilities [OR: 4.93 (3.93–6.20)] and a European or American versus a Spanish origin [OR: 1.62 (1.25–2.09) and 1.78 (1.30–2.44) respectively] (Table 1). The reasons why syphilis may be more common in males, especially MSM include several biological factors (i.e., anus mucosa is more susceptible to microinjury than vaginal mucosa), behavioral (multiple sexual partners or engaging in sex in higher risk settings), diagnostic (women may have infections without obvious symptoms, making early diagnosis difficult) and social stigma (increased vigilance of their sexual health).

**Table 1:** Univariate and multivariate data analysis of the impact of syphilis among people living with HIV.

		Syphilis/Total (%)	OR (95% CI) Univariate^*^	p value	OR (95% CI) Multivariate^**^	p value
**Age group (years)**	< 20	39/75 (52.00)	1.41 (0.75-2.69)	0.286	1.27 (0.64-2.50)	0.494
	20-39	415/814 (50.98)	1.59 (1.00-2.54)	0.050	1.35 (0.82-2.22)	0.236
	40-59	303/720 (42.08)	1.11 (0.70-1.78)	0.656	1.15 (0.70-1.90)	0.585
	≥ 60	32/81 (39.51)	Ref		Ref	
**Sex**	Male	758/1,487 (50.98)	6.50 (4.31-9.81)	**< 0.01**	2.46 (1.55-3.91)	**< 0.01**
	Female	28/203 (13.79)	Ref		Ref	
**infection vulnerabilities**	MSM	654/1,107 (59.08)	4.93 (3.93-6.20)	**< 0.01**	3.65 (2.81-4.73)	**< 0.01**
	Others^***^	132/583 (22.64)	Ref		Ref	
**Geographical** **origin**	Immigrants	285/550 (51.82)	1.37 (1.12-1.68)	**< 0.01**	1.37 (1.10-1.71)^*^	**< 0.01**
	Locals	501/1,140 (43.95)	Ref		Ref	
**Geographical** **area**	Europe (Spain excluded)	165/295 (55.93)	1.62 (1.25-2.09)	**< 0.01**	1.35 (1.02-1.77)^**^	0.035
	Americas	106/182 (58.24)	1.78 (1.30-2.44)	**< 0.01**	1.77 (1.26-2.49)^**^	**< 0.01**
	Africa	13/68 (19.12)	0.30 (0.16-0.56)	**< 0.01**	0.70 (0.36-1.37)^**^	0.300
	Asia/Oceania	1/5 (20.0)	0.32 (0.04-2.86)	0.307	0.33 (0.03-3.30)^**^	0.344
	Spain	501/1,140 (43.95)	Ref		Ref	

* Model includes age, sex, infection vulnerabilities and geographical origin

** Model includes age, sex, infection vulnerabilities and geographical area of origin

*** Includes: HTSP: heterosexuals, PWID: persons who inject drugs and patients with no data or categorized as “other”.

The multivariate analysis included the different variables listed in Table 1 in two initial models: the first model included age, sex (male versus female), infection vulnerabilities and origin (local vs immigrant). In the second model, the origin variable was replaced by the more specific geographical area of origin of the immigrants, with Spain as the reference group. Male sex remained statistically significant for the risk of presenting with *T. pallidum* infection, with an OR of 2.46 (95%CI 1.55–3.91) as did the MSM transmission category, with an OR of 3.65 (2.81–4.73) compared to other transmission categories. In terms of origin, immigrants showed an OR of 1.37 (1.10–1.71), and when this variable was replaced in the second model by their geographical area of origin, a European origin (excluding Spain) showed an OR of 1.35 (1.02–1.77) and an American origin an OR of 1.77 (1.26–2.49). An African origin showed a non-significant OR of 0.70 (0.36–1.37). These data were used to explore the possible statistical interaction between region of origin and sex, as well as transmission category, in a third model. In this model, the OR for European origin [OR 1.41 (0.37–5.42)] and the OR for American origin [OR 0.72 (0.15–3.40)] were no longer statistically significant. Table 2 describes this possible interaction and shows that syphilis prevalence among persons of European origin (excluding Spain) was 55.93%, with a male sex frequency of 94.58% and MSM of 81.36%; the scores for an American origin were similar, with a male sex frequency of 90.11% and MSM of 68.13%. For an African origin on the other hand, the syphilis frequency was 19.12% with male sex and MSM frequencies of 51.47% and 13.24%, respectively.

**Table 2:** Descriptive analysis of PLWH by geographical area of origin.

		Spain	Europe (excluding Spain)	Americas	Africa	Asia
**Syphilis**	Yes	501 (43.95)	165 (55.93)	106 (58.24)	13 (19.12)	1 (20.00)
**Age group** **(years)**	< 20	66 (5.79)	1 (0.34)	4 (2.2)	4 (5.88)	0 (0.00)
	20-39	554 (48.60)	111 (37.63)	105 (57.69)	41 (60.29)	3 (60.00)
	40-59	464 (40.70)	159 (53.90)	72 (39.56)	23 (33.82)	2 (40.00)
	> 59	56 (4.91)	24 (8.13)	1 (0.55)	0 (0.00)	0 (0.00)
**Sex**	Male	1,006 (88.25)	279 (94.58)	164 (90.11)	35 (51.47)	0 (0.00)
**infection vulnerabilities**	MSM^*^	731 (64.12)	240 (81.36)	124 (68.13)	9 (13.24)	3 (60.00)
	HTS^**^	212 (18.60)	31 (10.51)	39 (21.43)	55 (80.88)	2 (40.00)
	PWID^***^	89 (7.81)	4 (1.36)	2 (1.1)	1 (1.47)	0 (0.00)
	Other/no data	108 (9.47)	20 (6.78)	17 (9.34)	3 (4.41)	0 (0.00)

* MSM: men who have sex with men

** HTS: Heterosexuals

*** PWID: Persons who inject drugs

In patients with syphilis/HIV coinfection, the presence and, in positive cases, titers of the nontreponemal RPR test were evaluated. The results are shown in Table 3.

**Table 3:** RPR titers in syphilis (positive treponemal test)/HIV coinfection).

RPR		N	%
Negative		367	50.91
Positive	1:1	49	6.79
	1:2	77	10.70
	1:4	56	7.83
	1:8	26	3.66
	1:16	62	8.62
	1:32	34	4.70
	1:64	24	3.39
	1:128	15	2.09
	1:256	6	0.78
	1:512	4	0.52

## Discussion

In theory, syphilis should be an ideal disease to eliminate as a public health problem, for several reasons: *(i)* it is not a zoonosis, as there is no known animal reservoir, *(ii)* diagnosis can be made with simple and inexpensive methods, and *(iii)* treatment is effective and simple [1]. As noted above, *T. pallidum* subsp *pallidum* is transmitted only between humans, mainly by sexual transmission. It can be induced experimentally by inoculating rabbits, which is useful for isolating the bacterium and for studying the pathogenesis of the disease [2]. The diagnosis of syphilis is based on clinical suspicion, direct microbiological studies and serology. In the presence of the clinical manifestations mentioned above, direct diagnostic methods such as microscopic examination (darkfield or direct fluorescence) or nucleic acid amplification techniques (i.e., PCR) can be used. However, these techniques are not readily available, and microscopy can yield false-positive results in certain sites (such as the oral cavity or the rectum) due to the presence of commensal treponemes [3]. In clinical practice therefore the microbiological diagnosis of syphilis (symptomatic or asymptomatic) is based on serology [1–3,46–49]. There are two types of serologic tests for infection: treponemal and reaginic, or nontreponemal, with important differences in their interpretation. Treponemal tests detect specific antibodies against protein antigens of the genus *Treponema* (e.g., *TpN47*, *TpN17*, *TpN15*) whereas reaginic tests detect antibodies that recognize both host and treponemal lipoidal antigens (a combination of cardiolipin, lecithin, and cholesterol). Combined use of treponemal and reaginic tests, using either the traditional or the reverse algorithm, or rapid tests (immunochromatography) aids in the interpretation of results. Treatment of syphilis is based on penicillin, with different formulations and doses depending on the stage of the disease, or doxycycline in specific cases. Resistance to these antimicrobials has not been reported [50].

In HICs (high-income countries), a progressive increase in syphilis has been noted in recent years, particularly among MSM, people living with HIV, and immigrants [51,52]. The aim of this study was to evaluate the seroprevalence of syphilis in asymptomatic persons in our geographical region, taking into account the epidemiologic characteristics mentioned above.

To study the seroprevalence in the healthy population, we evaluated the data from blood donors, in which those with the legally defined risk factors had been excluded. The results showed a mean prevalence of 0.25%, with no significant variation during the study period, which was clearly higher than the national average for Spain [53]. On the other hand, the seroprevalence in this population was approximately one third of that observed in the general population at the beginning of the study period, suggesting higher transmission in the community. When the seroprevalence data in blood donors were compared with those of other series, significant differences (ranging from 0.03% to 0.90%) were observed [54–62]. These differences could be attributed to a number of factors, such as, geographical area (e.g., northern Europe [58,59,62], the Mediterranean area [54,57], India [55,60,61] or China [56]), the date of the study [55,60], the serological method used (reaginic or treponemal, as well as specific technique) [57,58,61] and donor selection criteria, more specifically, replacement donors versus voluntary donors [55].

The seroprevalence of syphilis in people living with HIV was 46.51%. In our setting, this is on average more more than 150 times that of the blood donors. This prevalence is very high, and among the highest reported in the literature [63–67]. The prevalence of syphilis-HIV coinfection ranges from 2 to 60%, depending on the date of the study, the country and geographical region [63–67]. Although not observed in the present series, more recent studies show a marked increase in frequency [68,69].

The study of reaginic tests in coinfected patients in this series showed three different patterns: RPR negative (50.9%); RPR 1:1–1:8 (28.98%) and RPR > 1:8 (20.10%). There are several possible explanations for patients with RPR ≤ 1:8, including the natural decline of a *T. pallidum* infection acquired several years earlier, or the use of antimicrobials with activity against this bacterium. In this regard, although the treatment of choice for syphilis is penicillin, other commonly used antibiotics have activity, sometimes incomplete, against *T*. *pallidum* [70,71]. There are also several interpretations for patients with RPR > 1:8, such as recent infection or reinfection, which is common in these individuals [72–74]. Whatever the interpretation, it is assumed that RPR titers > 1:8 indicate active infection and that these patients pose a risk of disease transmission [75].

The multivariate analysis of factors influencing HIV-syphilis coinfection clearly shows an association with male sex and the MSM risk category, both of which are repeatedly documented in the reviewed literature [73,76,77]. The higher prevalence of syphilis is observed not only in HIV-infected MSM patients, but also in HIV-negative MSM patients evaluated in Pre exposure prevention programs [78,79]. Therefore, it is particularly important in the control of syphilis to adopt strategies that target MSM individuals, such as prevention of both infections before acquisition (i.e., Doxy-PreP, with its limitations) [80–82] and frequent monitoring for *T. pallidum* infection during HIV screening [83]. In addition, MSM patients include not only homosexuals but also bisexuals [84,85]. One third of MSM in the USA or China reported sexual relations with women, almost half in Peru, and about 80% in Russia [84]. On the other hand, in the study of MSM in the USA, 14.5% reported sexual relations with women and 22.3% with men who in turn had relations with both sexes [85]. Therefore, bisexual men facilitate contact between men and women and represent a bridge in the transmission and spread of syphilis.

However, the results of this series raise the question of the overall role of immigration in the seroprevalence of syphilis among people living with HIV in our setting. We observed two different patterns: *i)* European and Latin American immigrants, where the preponderance of males and MSM transmission was similar to that of the Spanish population, and *ii)* African immigrants, where there was a higher frequency of syphilis seroprevalence among females and a much lower prevalence among MSM.

The study of recently arrived migrants from Africa with HIV infection provides important information while avoiding confounding variables in this group related to the acquisition of *T. pallidum* infection associated with other factors (such as sex work or drug use). The data obtained indicate a higher seroprevalence compared to the local blood donor population (5.30% versus 0.25%), although there were no cases of active syphilis (RPR ≤ 1:8). These earlier data are maintained in the very recent study by our group of newly arrived Africans in the Canary Islands [86]. Seroprevalence data in recently arrived migrants from Africa are very scarce and range from 2.2 to 11.7% [18,22,25].

Limitations of our study include: *i)* failure to identify previous medical problems related to syphilis and/or its treatment due to recall or memory bias, especially among African immigrants; *ii)* the absence of information on sexual risk behaviors in blood donors and people living with HIV due to non-response bias; *iii)* recording blood donations, not number of donors, so that the results of treponemal testing were higher than the actual ones, and *iv)* the different study dates in some groups (i.e., undocumented African immigrants).

## Conclusions

**Fig 2:**
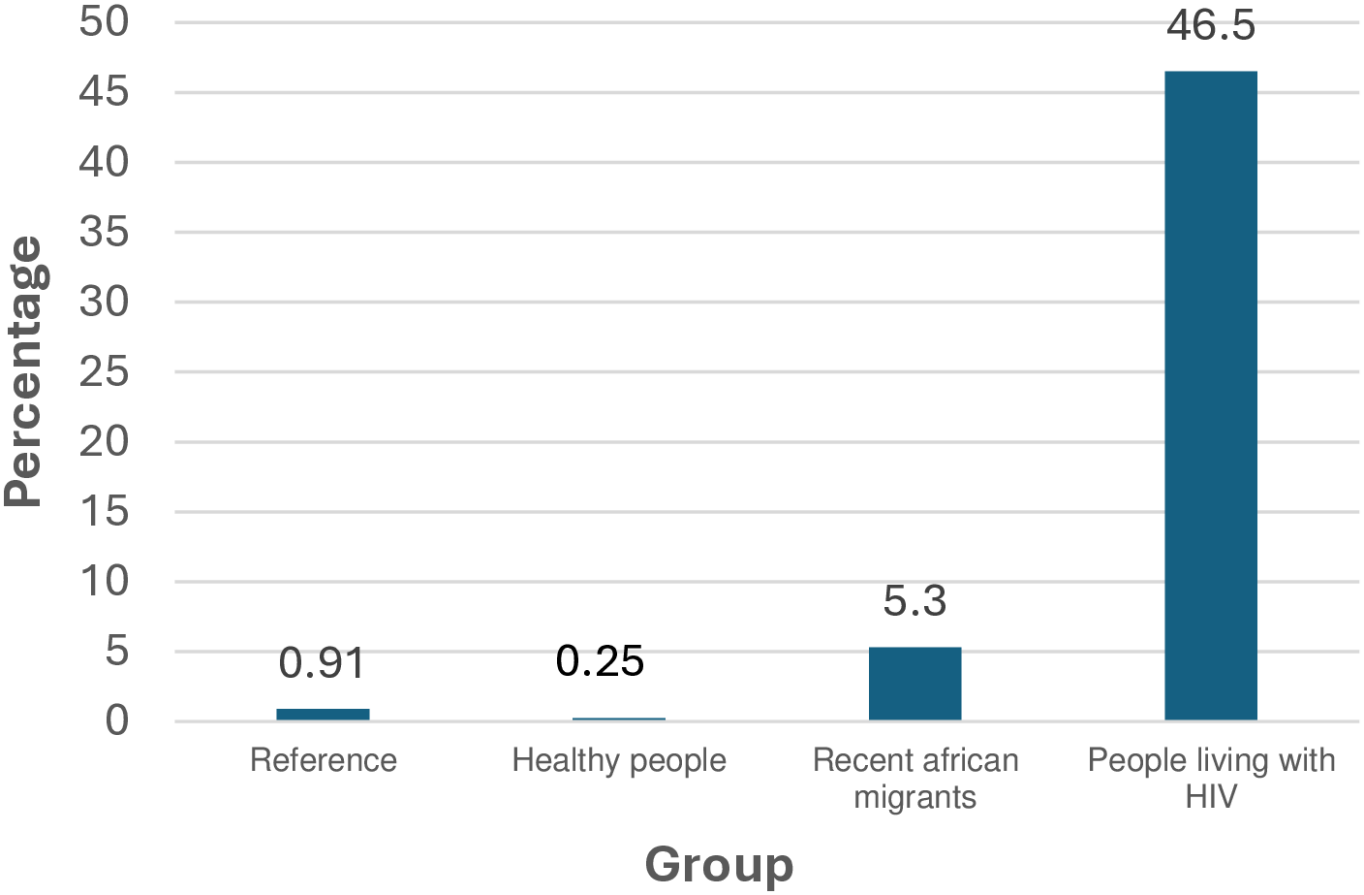
Seroprevalence of syphilis in the different groups (Gran Canaria) N (number of positive treponemal test)/ n (number of total group).

In conclusion, syphilis is a reemerging infection, and asymptomatic persons constitute a group that facilitates its transmission and dissemination. In our setting, seroprevalence is lowest in the blood donors, higher among recently arrived African immigrants and highest in people living with HIV, especially MSM (Fig 2). However, the presence of active syphilis is mainly restricted to MSM. This information is of relevance for the design of syphilis control strategies.

## Supporting information

S1 Table Prevalence of *T. pallidum* infection by group, year of study and gender. M/F: Male/Female.
(DOCX)

S2 Table Age and sex in the different study groups. M/F: Male/Female.
(DOCX)

S3 Table Additional microbiogical test in the study groups. (DOCX)

S1 Fig Geographic origin of undocumented migrants from Africa. Data include the total number of migrants from Africa, distinguishing between those from North Africa and Sub-Saharan Africa. The map has been created through the web https://d-maps.com/continent.php?num_con=1&lang=es.
(DOCX)
