# Supplementary material for: Treponema pallidum infection in asymptomatic persons: A puzzling scenario in the Canary Islands (Spain) (2001–2020)
Source: PLoS One. 2025 Jul 8;20(7):e0325073. doi: 10.1371/journal.pone.0325073 (PMC12237060; doi:10.1371/journal.pone.0325073)
Supplement: S1 Table — M/F: Male/Female. (DOCX) [file pone.0325073.s001.docx]

|  | **Blood donations** | | **Undocumented African migrants** | | **People living with HIV** | |
| --- | --- | --- | --- | --- | --- | --- |
| **Year** | **N** | **Positive** | **N** | **Positive** | **N** | **Positive** |
| **2001** | **Total: 28,505**  **M/F ratio: 1.88** | **Total: 24**  **M/F: 19/5** | **Total: 179**  **M/F ratio:3.37** | **Total: 4**  **M/F: 4/0** | **Total: 66**  **M/F ratio: 7.25** | **Total: 35**  **M/F: 32/3** |
| **2002** | **Total: 32,447**  **M/F ratio: 2.05** | **Total: 17**  **M/F: 15/2** | **Total: 215**  **M/F ratio: 9.75** | **Total: 9**  **M/F: 8/1** | **Total: 69**  **M/F ratio: 5.90** | **Total: 30**  **M/F: 28/2** |
| **2003** | **Total: 35,364**  **M/F ratio: 1.99** | **Total: 20**  **M/F: 14/6** | **Total:234**  **M/F ratio: 5.88** | **Total: 17**  **M/F: 15/2** | **Total: 55**  **M/F ratio: 5.88** | **Total: 20**  **M/F: 20/0** |
| **2004** | **Total: 39,643**  **M/F ratio: 1.89** | **Total: 32**  **M/F: 23/9** | **Total: 13**  **M/F ratio: 5.5** | **Total: 4**  **M/F: 4/0** | **Total: 79**  **M/F ratio: 6.90** | **Total: 44**  **M/F: 42/2** |
| **2005** | **Total: 43,807**  **M/F ratio: 1.82** | **Total: 55**  **M/F: 37/18** | **Total: -**  **M/F ratio: -** | **Total: -**  **M/F: -** | **Total: 54**  **M/F ratio: 9.80** | **Total: 26**  **M/F: 26/0** |
| **2006** | **Total: 49,853**  **M/F ratio: 1.74** | **Total: 89**  **M/F: 72/17** | **Total: -**  **M/F ratio: -** | **Total: -**  **M/F: -** | **Total: 42**  **M/F ratio: 5.00** | **Total: 15**  **M/F: 15/0** |
| **2007** | **Total: 51,177**  **M/F ratio: 1.77** | **Total: 186**  **M/F: 144/42** | **Total: -**  **M/F ratio : -** | **Total: -**  **M/F: -** | **Total: 49**  **M/F ratio: 2.77** | **Total: 22**  **M/F: 19/3** |
| **2008** | **Total: 53,772**  **M/F ratio: 1.70** | **Total: 284**  **M/F: 209/75** | **Total: -**  **M/F ratio: -** | **Total: -**  **M/F: -** | **Total: 60**  **M/F ratio: 5.67** | **Total: 36**  **M/F: 34/2** |
| **2009** | **Total: 56,427**  **M/F ratio: 1.66** | **Total: 251**  **M/F: 182/69** | **Total: -**  **M/F ratio: -** | **Total: -**  **M/F:** | **Total: 72**  **M/F ratio: 7.00** | **Total: 35**  **M/F: 35/0** |
| **2010** | **Total: 55,198**  **M/F ratio: 1.68** | **Total: 227**  **M/F: 163/64** | **Total: -**  **M/F ratio: -** | **Total: -**  **M/F: -** | **Total: 74**  **M/F ratio: 3.87** | **Total: 34**  **M/F: 33/1** |
| **2011** | **Total: 53,447**  **M/F ratio: 1.68** | **Total: 158**  **M/F: 123/35** | **Total: -**  **M/F ratio: -** | **Total: -**  **M/F: -** | **Total: 60**  **M/F ratio: 11.00** | **Total: 33**  **M/F: 31/2** |
| **2012** | **Total: 50,493**  **M/F ratio: 1.70** | **Total: 155**  **M/F: 115/40** | **Total: -**  **M/F ratio: -** | **Total: -**  **M/F:-** | **Total: 71**  **M/F ratio: 6.10** | **Total: 37**  **M/F: 35/2** |
| **2013** | **Total: 50,737**  **M/F ratio: 1.69** | **Total: 174**  **M/F: 125/49** | **Total: -**  **M/F ratio: -** | **Total: -**  **M/F: -** | **Total: 82**  **M/F ratio: 4.86** | **Total: 38**  **M/F: 35/3** |
| **2014** | **Total: 49,777**  **M/F ratio: 1.67** | **Total: 130**  **M/F: 89/41** | **Total: -**  **M/F ratio: -** | **Total: -**  **M/F: -** | **Total: 89**  **M/F ratio: 4.24** | **Total: 36**  **M/F: 35/1** |
| **2015** | **Total: 49,758**  **M/F ratio: 1.65** | **Total: 121**  **M/F: 91/30** | **Total: -**  **M/F ratio: -** | **Total: -**  **M/F: -** | **Total: 114**  **M/F ratio: 13.25** | **Total: 59**  **M/F: 57/2** |
| **2016** | **Total: 49,897**  **M/F ratio: 1.58** | **Total: 135**  **M/F: 95/40** | **Total: -**  **M/F ratio: -** | **Total:-**  **M/F: -** | **Total: 54**  **M/F ratio: 17.00** | **Total: 29**  **M/F: 29/0** |
| **2017** | **Total: 50,655**  **M/F ratio: 1.51** | **Total: 115**  **M/F: 84/31** | **Total: -**  **M/F ratio: -** | **Total:-**  **M/F: -** | **Total: 90**  **M/F ratio: 14.00** | **Total: 40**  **M/F: 39/1** |
| **2018** | **Total: 52,153**  **M/F ratio: 1.45** | **Total: 110**  **M/F: 87/23** | **Total: -**  **M/F ratio: -** | **Total: -**  **M/F: -** | **Total: 126**  **M/F ratio: 11.60** | **Total: 59**  **M/F: 58/1** |
| **2019** | **Total: 48,796**  **M/F ratio: 1.47** | **Total: 122**  **M/F: 90/32** | **Total: -**  **M/F ratio: -** | **Total: -**  **M/F: -** | **Total: 77**  **M/F ratio: 6.00** | **Total: 40**  **M/F: 38/2** |
| **2020** | **Total: 46,963**  **M/F ratio: 1.34** | **Total: 98**  **M/F: 78/20** | **Total: -**  **M/F ratio: -** | **Total: -**  **M/F: -** | **Total: 159**  **M/F ratio: 14.90** | **Total: 69**  **M/F: 69/0** |

**S1 Table. Prevalence of *T. pallidum* infection by group, year of study and gender.** M/F: Male/Female
