# Supplementary material for: Treponema pallidum infection in asymptomatic persons: A puzzling scenario in the Canary Islands (Spain) (2001–2020)
Source: PLoS One. 2025 Jul 8;20(7):e0325073. doi: 10.1371/journal.pone.0325073 (PMC12237060; doi:10.1371/journal.pone.0325073)
Supplement: S2 Table — M/F: Male/Female. (DOCX) [file pone.0325073.s002.docx]

| **Age** | **Blood donations** | **Undocumented African migrants** | **People living with HIV** |
| --- | --- | --- | --- |
| **18-24 years old** | **Total: 17,226**  **M/F ratio: 0.91** | **Total: 306**  **M/F ratio: 4.37** | **Total: 171**  **M/F ratio: 12.5** |
| **25-35 years old** | **Total: 103,065**  **M/F ratio: 1.13** | **Total: 288**  **M/F ratio: 8.97** | **Total: 458**  **M/F ratio: 9.41** |
| **36-45 years old** | **Total: 225,493**  **M/F ratio: 1.53** | **Total: 39**  **M/F ratio: 4.57** | **Total: 483**  **M/F ratio: 5.71** |
| **46-55 years old** | **Total: 315,889**  **M/F ratio: 2.10** | **Total: 8**  **M/F ratio: 1.33** | **Total: 303**  **M/F ratio: 5.89** |
| **56-65 years old** | **Total: 216,667**  **M/F ratio: 2.20** | **Total:-**  **M/F ratio:-** | **Total: 99**  **M/F ratio: 6.62** |
| **> 65 years old** | **Total: 70,529**  **M/F ratio: 2.03** | **Total:-**  **M/F ratio:-** | **Total: 28**  **M/F ratio: 8.33** |

**S2 Table. Age and sex in the different study groups.** M/F: Male/Female
