# Supplementary material for: Treponema pallidum infection in asymptomatic persons: A puzzling scenario in the Canary Islands (Spain) (2001–2020)
Source: PLoS One. 2025 Jul 8;20(7):e0325073. doi: 10.1371/journal.pone.0325073 (PMC12237060; doi:10.1371/journal.pone.0325073)
Supplement: S3 Table — (DOCX) [file pone.0325073.s003.docx]

|  | **Blood donations *** | **Undocumented African migrants** | **People living with HIV** |
| --- | --- | --- | --- |
| **HIV** | **0%** | **3.6%** | **100%** |
| **HBsAg** | **0%** | **10.9 %** | **2.9%** |
| **HCV** | **0%** | **1.1%** | **11.9%** |

**S3 Table. Additional microbiological test.** These data correspond to the patients included in this study. * In the general blood donor population from Canary Islands the prevalence per 100,000 donations of HIV ranges between 9 and 13, that of HBsAg between 11 and 17 and that of HCV between 9 and 11. (National Health System. Activity of Transfusion Centers and Services. Report 2022. [Sistema Nacional de Salud. Actividad de Centros y Servicios de Transfusión. Informe 2022]. Available at: https://www.sanidad.gob.es › publicaciones › docs. Accessed March 25, 2025.)
