# Supplementary material for: Treponema pallidum infection in asymptomatic persons: A puzzling scenario in the Canary Islands (Spain) (2001–2020)
Source: PLoS One. 2025 Jul 8;20(7):e0325073. doi: 10.1371/journal.pone.0325073 (PMC12237060; doi:10.1371/journal.pone.0325073)
Supplement: S1 Fig — Data include the total number of migrants from Africa, distinguishing between those from North Africa and Sub-Saharan Africa. The map has been created through the web https://d-maps.com/continent.php?num_con=1&lang=es. (DOCX) [file pone.0325073.s004.docx]

**Supplementary material**


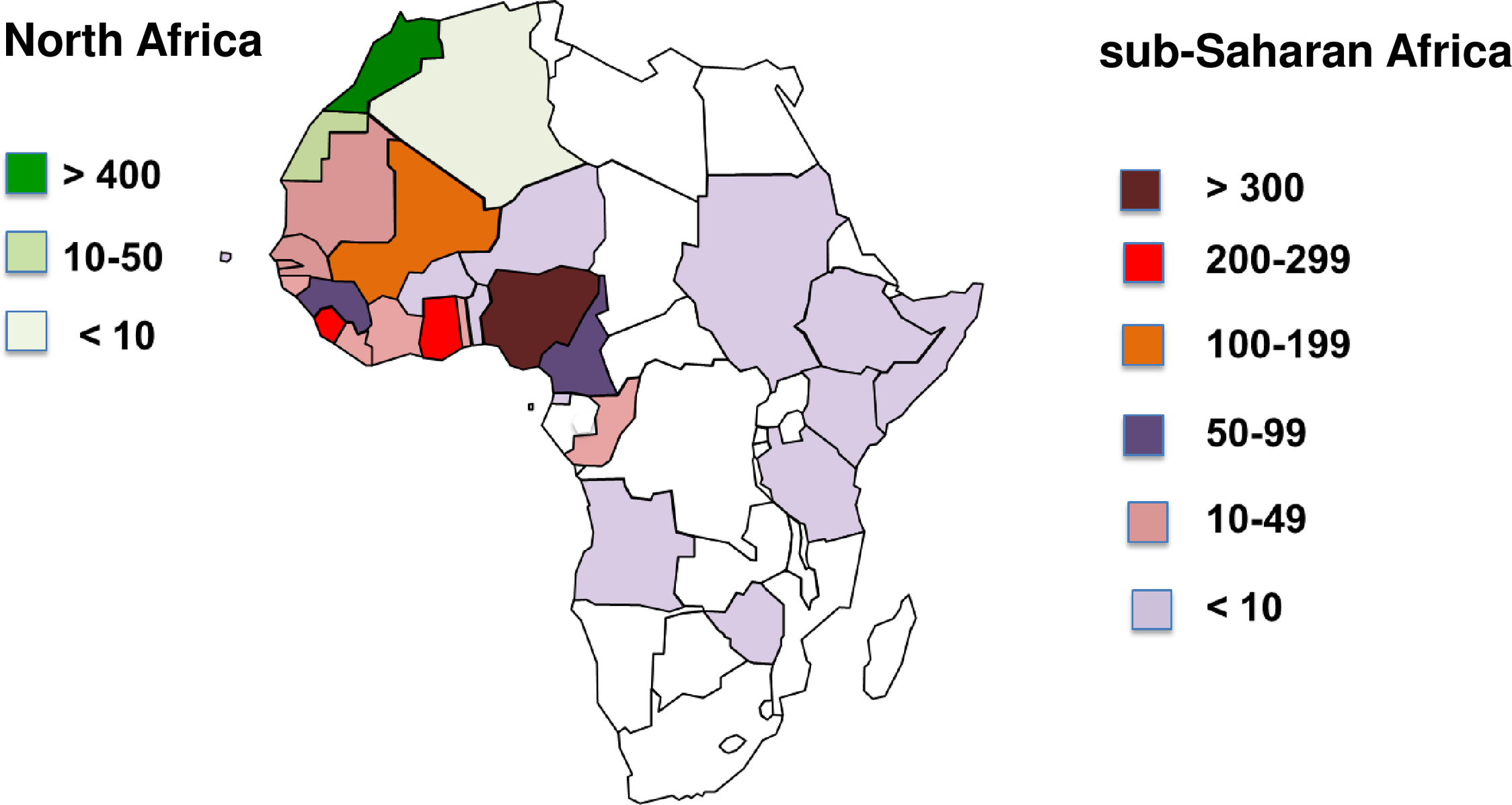


**S1 Figure. Geographic origin of undocumented migrants from Africa**

Data include the total number of migrants from Africa, distinguishing between those from North Africa and Sub-Saharan Africa. The map has been created through the web <https://d-maps.com/continent.php?num_con=1&lang=es> .
